# Enhancing context-aware SARS disorder management: a proposed multi-agent simulation framework with machine learning and bio-sensor data integration

**DOI:** 10.3389/fmedt.2026.1780837

**Published:** 2026-04-15

**Authors:** Abdullah, Zulaikha Fatima, Nida Hafeez, Muhammad Ateeb Ather, Rolando Quintero Tellez, Grigori Sidorov, Carlos Guzmán Sánchez-Mejorada, Miguel Jesús Torres Ruiz

**Affiliations:** 1 Computing Research Center (CIC), Instituto Politécnico Nacional (IPN), Mexico City, Mexico; 2 Department of Computer Science, Bahria University, Lahore, Pakistan; 3 Faculty of Allied Health Sciences, Superior University, Lahore, Pakistan

**Keywords:** Bayesian networks, context-aware system, knowledge representation, logic programming, no monotonic reasoning, probability model construction, reasoning under uncertainty, wireless sensor network

## Abstract

In this work, SARS disorder denotes a generic acute severe respiratory distress condition characterized by abnormal respiratory rate, oxygen saturation, fever, and cardiovascular stress indicators, and does not represent a COVID-19 diagnostic system. Our research aims at analyzing a context-aware SARS disorder management system through the implementation of a multi-agent simulation framework using the NetLogo setting. The system relies on the use of interacting agents as well as non-monotonic, context-sensitive reasoning to reduce uncertainty and deal with the possible inconsistencies that happen due to biosensor recordings. A knowledge-based inference component is the combination of physiological sensor outputs and domain specific contextual data to assist in making informed decisions. The research involved the use of several machine-learning classifiers, that is, Naïve Bayes, Multinomial Naïve Bayes, Decision Table, Logistic Regression, and Sequential Minimal Optimization (SMO) so as to evaluate their appropriateness in being incorporated into the developed structure. To measure the system performance, standard evaluation measures were used such as True Positive (TP), False Positive (FP), Precision, Recall, F-Measure, Matthews Correlation Coefficient (MCC), Receiver Operating Characteristic (ROC) curve, and the Precision-Recall curve (PRC). The framework includes a list of physiological, environmental, and contextual variables, such as electrocardiographic parameters, heart-rate parameters, blood-pressure parameters, arterial oxygen saturation parameters, core body temperature, room temperature, the past history of the patient, and parameters that relate to alerts. The classification task is to produce probabilistic forecasts that help to define whether a patient should be alerted or clinical staff members informed in order to facilitate context-specific healthcare response.

## 1 Introduction

Uncertainty is an essential component of life that influences decision-making across diverse domains, including healthcare, finance, and engineering (1, 2). In healthcare, uncertainty arises from complex human physiology, variability in patient responses, incomplete sensing, and dynamic environmental conditions. These challenges make accurate and timely decision-making particularly difficult in-patient monitoring scenarios, where real-time responses can substantially affect patient outcomes. Consequently, the role of probabilistic reasoning and context-aware systems has become increasingly important (3).

Probability provides a quantitative framework for representing uncertainty and supporting rational decision-making under incomplete information. Concepts such as conditional probability, joint probability, and prior posterior updating allow systems to revise beliefs as new evidence becomes available (4, 5). These principles are especially relevant in intelligent healthcare applications, where physiological measurements are noisy, partially observed, and continuously evolving.

Within multi-agent systems, autonomous agents frequently operate with incomplete or uncertain data and must rely on probabilistic reasoning to coordinate decisions effectively (6, 7). While some intelligent systems employ non-probabilistic classifiers, probabilistic representations are often incorporated to enhance decision confidence and interpretability. For instance, Support Vector Machines (SVMs) are fundamentally non-probabilistic classifiers that aim to maximize class separation margins (8). Nevertheless, probabilistic calibration techniques such as Platt scaling and isotonic regression enable SVM decision values to be transformed into posterior probability estimates (9). These developments highlight the broader importance of probabilistic outputs in safety-critical applications, even when classical learning models are employed.

Context awareness plays a critical role in modern healthcare systems, particularly in patient monitoring environments where conditions such as patient mobility, sensor reliability, and environmental factors continuously change. Failure to account for contextual variation can introduce uncertainty and experimental error, especially under dynamic conditions such as temperature fluctuations or resource constraints (10, 11). Integrating context-awareness with intelligent decision-making therefore remains a key research challenge.

Multi-agent systems offer a promising solution by enabling distributed sensing, reasoning, and decision-making across autonomous entities while explicitly managing uncertainty through probabilistic methods (12, 13). Despite advances in this area, the adoption of context-aware healthcare systems remains limited. Many existing solutions require clinicians to adapt to rigid workflows that do not align with real-world clinical practice, reducing usability and trust (14, 15).

Severe Acute Respiratory Syndrome (SARS), a potentially fatal respiratory infection caused by a coronavirus, emerged in 2002 and underscored the global risks posed by rapidly spreading respiratory conditions. The unpredictable and zoonotic nature of such diseases continues to motivate research into intelligent monitoring systems that can support early detection and adaptive response strategies (16–18). In parallel, advances in networking, wireless communication, and sensor technologies have enabled continuous health monitoring across diverse clinical settings, including patient care, eldercare, and rehabilitation (19, 20).

Despite their theoretical promise, many context-aware and probabilistic healthcare frameworks remain insufficiently validated in practical settings. These systems typically rely on autonomous agents that combine contextual information and probabilistic reasoning to support decision-making under uncertainty (21–23). Bridging the gap between conceptual frameworks and deployable healthcare solutions therefore remains an open research problem.

In this study, the term SARS disorder refers to a generalized acute severe respiratory syndrome characterized by abnormal respiratory rate, oxygen saturation, fever, and cardiovascular stress markers. The proposed framework is not intended as a COVID-19 diagnostic system, but rather as a generic respiratory distress monitoring and early-warning platform applicable to SARS-like conditions. Motivated by the challenges outlined above, this research proposes a context-aware, probabilistic, multi-agent framework for intelligent patient monitoring. The framework integrates probabilistic machine learning with agent-based simulation using WEKA and NETLOGO (24), enabling robust handling of missing data, uncertainty, and dynamic contextual changes. Its effectiveness is evaluated through extensive experimentation across probabilistic reasoning strategies, machine learning models, and decision-making scenarios (25). Beyond performance evaluation, the study also identifies implementation challenges and outlines directions for future research to support real-world deployment (26).

### 1.1 Scientific contribution

This article proposes advancements in context-aware SARS disorder management. The main scientific contributions of the authors through this work are as follows:

- Proposed a pioneering non-monotonic context-aware system that integrates multi-agent simulation, machine learning algorithms, and bio-sensor data to effectively minimize data uncertainty and inconsistency, paving the way for more accurate patient monitoring.
- Developed a comprehensive multi-agent system simulation in NETLOGO, allowing agents to collaboratively make probabilistic decisions while handling uncertainty through Bayesian reasoning. This simulation framework is unique in its ability to manage SARS disorder in real-time using distributed agents.
- Conducted a groundbreaking comparative analysis of five machine learning algorithms (Naïve Bayes, Multinomial Naïve Bayes, Decision Table, Logistic Regression, and SMO) using a rich set of performance metrics like True Positive (TP), False Positive (FP), Precision, Recall, F-Measure, Matthews Correlation Coefficient (MCC), Receiver Operating Characteristic (ROC), and Precision-Recall Curve (PRC). The study offers insights into optimizing algorithm selection for minimizing data uncertainty.
- Introduced an advanced framework that seamlessly integrates probabilistic reasoning through Bayesian Networks and knowledge base reasoning to accurately assess patient conditions. This unique approach prioritizes decision-making for optimal healthcare management.
- Pioneered the incorporation of diverse bio-sensor and ambient data (ECG, heartbeat, room temperature, blood pressure, blood oxygen saturation) with domain context knowledge, providing a comprehensive, adaptable, and real-time monitoring solution.
- Validated the proposedprobabilistic framework using simulations in NETLOGO and Weka environments. The evaluation framework, utilizing a broad set of performance metrics, provides actionable insights into the reliability and efficacy of the system.
- Proposed forward-looking recommendations for future work, emphasizing advanced machine learning techniques, dynamic context adaptation, personalized healthcare, and human-AI collaboration. Identified challenges and opportunities in implementing the framework, laying a unique foundation for future contributions in context-aware SARS disorder management.

These contributions collectively aim to deliver an effective and adaptable healthcare monitoring system that leverages context-awareness, multi-agent reasoning, and probabilistic decision-making to enhance SARS disorder management. Section 1 gives a comprehensive introduction to the research topic. Section 2 provides an overview of existing research related to the topic and offers a comparison of several methodologies that have been used. Section 3 outlines the methodologies employed in carrying out the research. This section provides an explanation of the dataset, the suggested system, the study methodology, and the entire workflow. Section 4 outlines the key findings of the study. Section 5 provides a concise overview of the primary results of the study and examines their significance. An overview of possible avenues for future research is also given.

## 2 Literature review

For patient monitoring, Hossain Kordestani's Hapicare healthcare system incorporates probabilistic reasoning, IoT sensors, and ontologies. It improves decision-making and diagnostics by utilizing Bayesian networks, rule engines, and humanoid robots. The ultimate goal is to improve healthcare quality while decreasing costs through context- aware monitoring and personalized care (27, 28). Carlos Rojas-Guzman proposed a Bayesian network-based approximation approach for complicated network inference. The evaluation took into account inference time and diagnostic accuracy (29).

Todor Dimitrov presented a smart home architecture based on Bayesian networks and ontologies, which would allow for informed judgements and error detection in system monitoring (30). Pol Olivella-Rosell provided a probabilistic model for electric car charging, which improved the accuracy of demand prediction (31). Yang Liu's research combines probabilistic logic and multi-agent systems to improve decision-making in uncertain contexts through simulation and model validation. Future research will focus on agent communication and knowledge operators (32). Moazam Ali created a programmed composition technique for a flexible agent-based load monitoring system that includes fuzzy-based load adjustment. The fuzzy integrated model outperformed the others in terms of accuracy and speed (33).

Pol Olivella-Rosell presented the pRB-ATL logic for defining agent alliance features under resource constraints and probabilistic behavior. The pRB-ATL model-checking technique enables the verification of many subjective features in real-world systems (34). Hoang Nga Nguyen developed a theory for analyzing coalitions with limited resources that combines probabilistic and non-deterministic behavior. The pRB-ATL model verification technique allows for the representation of interesting agent alliance characteristics (35).

Shrinivasan Patnaikuni proposed the Multi-Entity Bayesian Networks (MEBN) hypothesis, which blends first-order logic expressivity with probabilistic reasoning. MEBN depicts connected random variables and associated probability distributions graphically (36). Xin Li and José-Fernán Martnez focused on context-awareness in underwater robots, developing a probabilistic reasoning-based context-aware system employing Multi-Entity Bayesian Networks (MEBN) (37). Daniele Riboni created an unsupervised approach for comprehending complicated activities of daily living (ADLs) through the use of semantic links and sensor events. The method overcomes the limits of supervised learning by recognizing interleaved activities (38). Paulo Games suggested a revolutionary strategy to uncertainty management in the semantic web based on probabilistic thinking (39). Dagmawi Neway Mekuria proposed a probabilistic multi-agent architecture for intelligent homes, with the goal of improving agent reasoning in unpredictable environments (40).

Pedro Oliveira suggests an approach to dealing with ambiguity in the semantic web. To deal with uncertainty, he utilizes occurrence facts and probabilistic reasoning. His method entails employing Markov logic to learn and reason about uncertainty in OWL-DL ontologies (39). In the larger framework of the Semantic Web, the goal is to allow the open and autonomous interchange of structured knowledge. This platform has seen a variety of techniques to dealing with un- certainty, including the extension of Semantic Web languages to include probabilistic, possibilistic, or fuzzy logics. These modifications improve the system's capacity to deal with unclear data (41). Markov logic is an approach that combines first-order logic and probabilistic graphical models, namely Markov networks. This method is useful for de- scribing and reasoning about ambiguous data. It has uses in disciplines such as medicine, where it aids in diagnosis, and product development, where it aids in managing profound uncertainty (42).

Probability and Bayesian reasoning are important in providing a rational framework for making decisions in the face of uncertainty. These techniques provide efficient data representation and computation. Probabilistic reasoning assists in making educated decisions based on uncertain facts in sectors ranging from healthcare to product design (43, 44) (Table 1).

**Table 1:** Comparison of techniques.

Article	Naïve Bayes	Rules	Simulation of multi-agent system	Context aware data	Random forest	Probability of decision	SMO	Logistic regression	Multi Naïve Bayes	Decision table
Mileo et al. (45)	No	Yes	Yes	Yes	No	No	No	No	No	No
Ngo and Haddawy (46)	Yes	Yes	No	Yes	No	No	No	No	No	No
Kawai et al. (47)	Yes	No	No	Yes	Yes	Yes	No	No	No	No
Proposed	Yes	Yes	Yes	Yes	Yes	Yes	Yes	Yes	Yes	Yes

### 2.1 Research gap

Based on the context and motivation outlined in Section 1.1 and the current research summarized in Section 2.1, several gaps in the existing body of knowledge have been identified:

- Limited Integration of Probabilistic Reasoning in Context-Aware Systems: While probabilistic reasoning plays a significant role in decision-making under uncertainty, current context-aware healthcare systems have not fully integrated advanced probabilistic models like Bayesian Networks and knowledge base reasoning to manage multi-sensor bio-data effectively. Therefore, there is a need for a comprehensive framework that can minimize uncertainty and inconsistency in SARS disorder management.
- Inadequate Comparative Analysis of Machine Learning Algorithms: Existing research lacks a thorough comparative analysis of machine learning algorithms in a context-aware healthcare setting, particularly for SARS disorder management. Studies primarily focus on individual algorithms or compare only a limited subset, leaving a gap in understanding the optimal choice of algorithms based on performance metrics like precision, recall, F-measure, MCC, ROC, and PRC.
- Insufficient Exploration of Multi-Agent Systems in Healthcare: Although multi-agent systems offer the potential to handle dynamic healthcare environments, there is insufficient research on their application in SARS disorder management. The existing literature does not provide an in-depth evaluation of how multi-agent systems can be effectively simulated to improve collaborative decision-making and reduce uncertainty.
- Lack of Comprehensive Bio-Sensor Data Integration: Previous works have incorporated bio-sensor data inconsistently, often focusing on a limited set of parameters. A more comprehensive approach that integrates ECG, heartbeat, room temperature, blood pressure, and other bio-sensor data with domain context knowledge is required to build a more adaptable and accurate monitoring system.
- Absence of Framework Validation through Simulation-Based Testing: There is a noticeable absence of framework validation through comprehensive simulation-based testing in healthcare management. A need exists for a simulation framework that can rigorously validate a proposed context-aware system in a multi-agent environment, providing actionable insights into its reliability and efficacy.
- Lack of Forward-Looking Recommendations for Enhancement: Although some studies have discussed the challenges in healthcare management, detailed forward-looking recommendations for enhancing non-monotonic context-aware systems are largely absent. Research often overlooks the potential of dynamic context adaptation, personalized healthcare, and human-AI collaboration in future framework improvements.

These research gaps justify the need for a novel context-aware SARS disorder management system, as proposed in Section 2.1. The proposed system uniquely integrates multi-agent simulation, machine learning algorithms, and bio-sensor data to enhance performance and minimize data uncertainty.

## 3 Methodology

**Figure 1:**
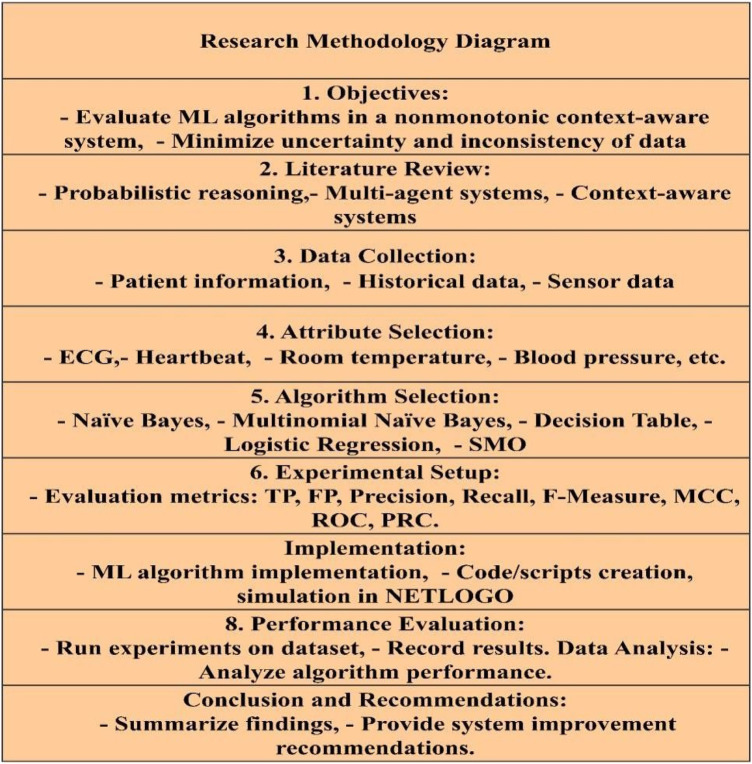
Research methodology.

The research methodology employs ML algorithms in a context-aware system to enhance data reliability as shown in Figure 1. A literature review explores probabilistic reasoning, multi-agent systems, and context-awareness. Data collection integrates patient, historical, and sensor data for dataset creation. Attribute selection targets ECG, heartbeat, temperature, and blood pressure.

ML algorithms like Naïve Bayes and Logistic Regression are chosen for their efficacy. Evaluation metrics include TP, FP, precision, recall, F-measure, MCC, ROC, and PRC. Implementation involves coding and simulation on NETLOGO. Performance evaluation assesses algorithm performance using collected data. Analysis focuses on established metrics. The study concludes with findings summary and improvement recommendations.

Context-awareness is modeled as a dynamic state vector comprising physiological signals, environmental conditions, and patient history. Context updates occur at each simulation tick based on sensor inputs and probabilistic inference. Rule-based reasoning operates at the agent level, while probabilistic outputs from ML classifiers influence alert states. To address scalability, rules are modularized by context category (physiological, temporal, environmental), enabling incremental extension without exponential rule growth.

### 3.1 Research design

**Figure 2:**
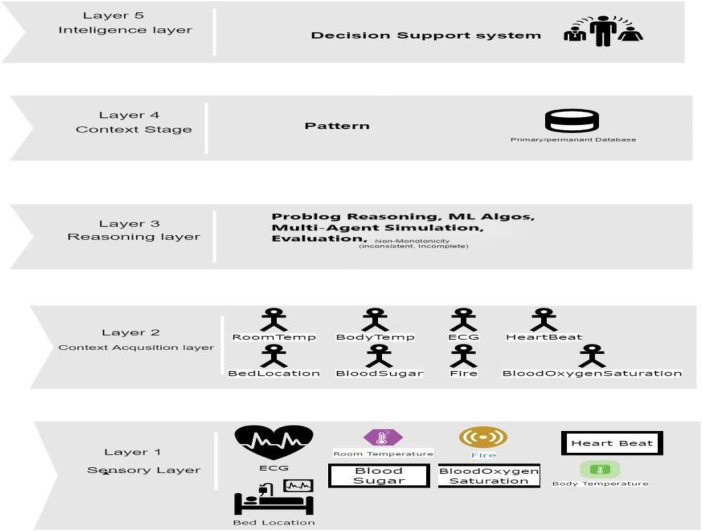
Methodology flow.

The proposed study technique has a tiered structure that integrates context acquisition, data input, semantic reasoning, and decision-making for optimal healthcare management. The technique is intended to extract insights from biosensors, ambient sensors, and patient data, and is built around the following layers as shown in given below Figure 2:

- Layer 1—Data Input Layer: This layer serves as a gateway for sensory data emanating from biosensors and ambient sensors. It incorporates patient-related context, such as history information and physical location, smoothly.
- Layer 2—Context Acquisition: Contextual information, including patient history and environmental data, is rigorously gathered here. To derive meaningful insights from the combined data, this layer combines Bayesian Network models and rule reasoning techniques.
- Layer 3—Semantic Reasoning: Digging further, the semantic reasoning layer analyses contextual data and per- forms knowledge queries. It employs probabilistic reasoning approaches to uncover useful patterns, insights, and probabilities related to patient well-being and environmental dynamics.
- Layer 4—Decision-Making and Reporting: At the apex, the technique leverages the knowledge and probability developed in previous levels to drive educated judgements. This approach is aided by domain-specific ontologies and expert knowledge. Furthermore, based on the analyzed information, the system has the authority to conduct actions such as requesting an ambulance.

Components and Processes: The technique integrates bio-sensor and environmental sensor inputs to provide a comprehensive patient health and environmental monitoring system. Temporal management and historical data analysis make it possible to identify important health patterns. Bayesian Network models and rule-based reasoning provide light on the probabilistic aspects of patient health and possible disease risks. Context-driven decision-making is aided by customized domain ontologies and expanded knowledge sources. Intelligent reports and timely warnings are created automatically, simplifying interventions and replies. Outcome: This study methodology's ultimate purpose is to revolutionize healthcare management. It accelerates healthcare choices into an era of heightened accuracy and speed by embracing a complete, context-sensitive strategy that incorporates bio-sensor data, environmental insights, patient histories, and probabilistic reasoning. This comprehensive strategy aims to improve patient well-being while also raising safety requirements in healthcare settings.

### 3.2 Dataset

A sample of this dataset was obtained by the University Accredited Hospital known as “UOL Teaching Hospital,” confirming data quality and trustworthiness. Data was obtained from the official database software “Oracle” and the hospital was given permission to access the data on 07/05/2022. Patients: This dataset contains data on up to 300 patients. Medical Sensors: It contains information from 20 different types of medical sensors. Choice Class: A choice class property with values ranging from 0 to 500 is present. History Data: The collection includes up to 300 occurrences of history data for patient records. Room Temperature: Room temperature data is collected, with values surpassing 50 °F. Blood Oxygen Saturation: Levels of blood oxygen saturation range from 15% to 19%. Body Temperature: The data for body temperature varies from 98 °F to 105 °F. ECG (Electrocardiogram): ECG values ranging from 80 to 120 are obtainable. Blood Pressure: The normal range for blood pressure is 60–175 mmHg. Heart Rate: Heart rate data ranges from 60 to 100 beats per minute. This dataset, gathered from the prestigious “UOL Teaching Hospital,” provides a comprehensive col- lection of healthcare and patient-related data, providing data quality and reliability for a variety of analytical and healthcare applications.

The dataset used in this study was constructed to support both simulation-based evaluation and external validation of the proposed context-aware healthcare framework. The simulation dataset comprises 1,000 virtual patients, each represented by 120 physiological records, corresponding to sequential monitoring intervals within the agent-based environment. Each record includes multivariate physiological attributes such as heart rate, ECG-derived features, body temperature, systolic and diastolic blood pressure, and oxygen saturation.

Patients were categorized into two clinically motivated classes: Normal and SARS-risk, where the latter represents a generalized severe respiratory distress condition. The class distribution was maintained at approximately 60% Normal and 40% SARS-risk to reflect moderate class imbalance commonly observed in clinical monitoring scenarios and to enable robust evaluation of classifier sensitivity and specificity.

Physiological value ranges used for dataset construction and simulation parameterization were derived from established clinical reference standards and were reviewed and validated by certified physicians with experience in respiratory and critical care medicine. This clinical oversight ensured that all simulated vital signs including heart rate, blood pressure, body temperature, and oxygen saturation remained within medically plausible bounds representative of both normal and respiratory distress conditions.

#### 3.2.1 Data preprocessing

Prior to model training and evaluation, all physiological attributes underwent standardized preprocessing procedures. Missing values were handled using forward-fill strategies for time-consistent signals and mean imputation for isolated missing entries. To ensure comparability across heterogeneous physiological scales, all features were normalized using Min–Max scaling.

Physiological noise and transient artifacts were reduced through moving-average smoothing, improving signal stability while preserving clinically relevant trends. These preprocessing steps were applied consistently across both the simulation dataset and the external validation dataset to ensure fair and reproducible performance assessment.

All patient data used to parameterize the simulation environment were synthetically generated and anonymized, based on clinically reported physiological ranges and established medical guidelines. No identifiable patient information was accessed or processed. External validation employed the publicly available clinical database, which contains fully de-identified intensive care unit records and is distributed under approved data use agreements. Consequently, institutional ethical approval was not required for this study.

### 3.3 Proposed system

The Platform is an intelligent monitoring and support system that is aimed to give patients with personalized treatment for their medical issues. It consists of several components that work together to achieve accurate diagnoses and effective decision-making. The perception layer is in charge of gathering data from many sources, such as sensors, self- assessments, and professional tests. This layer collects real-time data about patients' health and activity.

The data conversion layer connects the acquired data to the standardized ontology (48, 49). It is in charge of mapping and translating raw data into an ontology-compatible format. This guarantees that the acquired data can be processed and used properly inside the platform. Bayesian Belief Network (BBN): The Platform's BBN component maintains medical information and supports probabilistic reasoning. Bayesian belief networks, which are graphical models that depict connections between variables and their probability, are used. Based on observable causes and symptoms, the BBN assesses the probability of medical disorders, allowing for probabilistic diagnosis and decision-making. Rule Engine: The rule engine is an important component of the Platform.

It communicates with the BBN, gathers data from the physical environment, and makes choices based on established criteria. Based on fresh information, the rule engine activates the BBN graphs, offers more assessments or measurements in situations of doubt, chooses suitable reactions based on probability thresholds, and verifies the efficacy of prescribed actions. The rule engine solution is Smart Rules, a high-level rule language linked with ontologies. The agent component serves as a liaison between the Platform and the patients. It communicates with patients by transmitting system- triggered activities, requesting measures or assessments, and receiving replies. The agent can take the shape of a smartphone app, a web portal, or even a physical robot.

#### 3.3.1 Naïve Bayes classification

The equation for Naive Bayes classification can be expressed as follows:

Given a dataset with features $x_{1},x_{2},\ldots ,x_{n}$ and a class label *Y*, we want to calculate the probability of a specific class $C_{k}\;$ given the feature values as shown in Equation 1:

$$\left(\frac{C_{k}}{x_{1},x_{2},\ldots ,x_{n}}\right)=\frac{\frac{P(C_{k}).(x_{1},x_{2},\ldots ,x_{n})}{C_{k}}}{\left(x_{1},x_{2},\ldots ,x_{n}\right)}$$ (1)

In the context of Naive Bayes, we make the simplifying assumption that the features *X*_1_, *X*_2_,…, *X_n_* are conditionally independent given the class *C_k_*. This assumption is why it's called “Naive.” With this assumption, we can rewrite the equation as According to Bayes' Theorem, this can be calculated as shown in Equation 2:

$$\left(\frac{C_{k}}{x_{1},x_{2},\ldots ,x_{n}}\right)=\frac{(C_{k}).\left(\frac{x_{1}}{C_{k}}\right).P\left(\frac{x_{2}}{C_{k}}\right)\ldots P\left(\frac{x_{n}}{C_{k}}\right)}{\left(x_{1}).\;P(x_{2})\ldots P(x_{n}\right)}$$ (2)

Now, let's break down the terms, *P*(*Ck*) is the prior probability of class $(C_{k}).\left(\frac{x_{1}}{C_{k}}\right).P\left(\frac{x_{2}}{C_{k}}\right)\ldots P\left(\frac{x_{n}}{C_{k}}\right)$ are the conditional probabilities of each feature given class *Ck*. These can be estimated from the training data. $\left(x_{1}).\;P(x_{2})\ldots P(x_{n}\right)$ are the marginal probabilities of each feature may also be computed using the training data. To make a classification choice, we compute the aforementioned probability for each class and select the class with the highest probability as the projected class. In practice, several assumptions and approaches may differ depending on the Naive Bayes version (e.g., Gaussian Naive Bayes, Multinomial Naive Bayes) and the type of the data (e.g., continuous, discrete, or text data) (50).

#### 3.3.2 Multinomial naive Bayes

Multinomial Naive Bayes is a text classification algorithm that uses features to indicate the frequency of words or tokens in a document. The Multinomial Naive Bayes classification equation is derived from the more generic Naive Bayes formula, with the assumption that the features have a multinomial distribution.

Here's the Equation 1 Given a dataset with features $x_{1},x_{2},\ldots ,x_{n}$ (representing word frequencies) and a class label *Y*, we want to calculate the probability of a specific class $C_{k}$ given the feature values (word frequencies) In the context of Naive Bayes, we make the simplifying assumption that the features $x_{1},x_{2},\ldots ,x_{n}$ are conditionally independent given the class $C_{k}$. This assumption is why it's called “Naive.” With this assumption, we can rewrite the equation as According to Bayes' Theorem, this can be calculated as:

Now, let's break down the terms $P(C_{k})\;$ is the prior probability of class $(C_{k}).\left(\frac{x_{1}}{C_{k}}\right).P\left(\frac{x_{2}}{C_{k}}\right)\ldots P\left(\frac{x_{n}}{C_{k}}\right)\;\;$ are the probabilities of observing each word or token (feature) given class *Ck*. These probabilities are usually estimated from the training data by counting how often each word appears in documents of that class. $\left(x_{1}).P(x_{2})\ldots P(x_{n}\right)$ are the marginal probabilities of each word or symbol, which may also be computed using training data. To make a classification choice, we compute the aforementioned probability for each class and select the class with the highest probability as the projected class. Multinomial Naive Bayes is often used for text classification tasks such as spam detection or sentiment analysis, where the features in the documents are generally word counts or phrase frequencies. A decision table is a graphical depiction of a decision-making process that aids in determining the conclusion depending on many input criteria. It is frequently used in decision support and rule-based systems. In contrast, logistic regression is a statistical model used for binary or multiclass categorization (50).

#### 3.3.3 Decision table

The outcome of a decision table is decided by the combinations of input circumstances and their related actions or decisions. The underlying equation is a logical representation rather than a mathematical one. It may be stated as follows as shown in Equation 3:

$$\begin{array}{r} \mathrm{IF}\;(\mathrm{Conditio}n_{1}\;\mathrm{AND}\;\mathrm{Conditio}n_{2}\;\mathrm{AND}\;\;\ldots \ldots .\mathrm{AND}\;\mathrm{Conditio}n_{n})\\ \;\mathrm{then}\;\mathrm{Action}\; \end{array}$$ (3)

Where: $\mathrm{Conditio}n_{1}\;\mathrm{AND}\;\mathrm{Conditio}n_{2}\;\mathrm{AND}\;\mathrm{ldots}\ldots \ldots .\mathrm{AND}\;\;\mathrm{Conditio}n_{n}$ are the input conditions. Action is the decision or outcome associated with that combination of conditions. A decision table may have multiple rules like this, each specifying different conditions and corresponding actions (50).

#### 3.3.4 Logistic regression

Logistic regression is a mathematical model used for binary or multiclass classification problems. In binary logistic regression, the equation can be expressed as shown in Equation 4:

$$P(Y\;=\;1)=\frac{1}{e^{-(b_{0}+b_{1}x_{1}+b_{2}x_{2}\cdots b_{2}x_{n})}}$$ (4)

Where: *P*(*Y* = 1) is the probability of the dependent variable (*Y*) being in class 1. *e* is the base of the natural logarithm. *b*_0_ is the intercept. $b_{1},b_{2}\cdots b_{n}\;$ are the coefficients associated with the independent variables $x_{1},x_{2},\ldots ,x_{n}$ are the values of the independent variables. We have many equations in multiclass logistic regression, one for each class, and we use a softmax function to get the probabilities for each class. Based on the values of the independent variables, this equation estimates the likelihood that an instance belongs to class 1 (in binary logistic regression). If the likelihood exceeds a certain threshold (often 0.5), the instance is assigned to class 1; otherwise, it is assigned to class 0. In a classification problem, decision tables reflect decision-making logic, whereas logistic regression equations model the likelihood of class membership. They perform distinct functions and are employed in various circumstances (50).

#### 3.3.5 SMO (sequential minimal optimization)

SMO (Sequential Minimal Optimization) is a training technique for support vector machines (SVMs). SMO's purpose is to efficiently optimize the SVM's dual issue. The technique picks and optimizes pairs of Lagrange multipliers (values) repeatedly in order to maximize the margin between classes while fulfilling specific restrictions. Here's a simplified explanation of SMO algorithm, complete with equations.

Initialize and set the threshold *b*: Begin by setting the Lagrange multipliers (values) and the threshold (*b*). Equation 2 Select two *α* values to optimize: In each iteration, SMO selects two Lagrange multipliers $\left(a_{i}\;\mathrm{and}\;a_{j}\right)$ to optimize. These *α* values should satisfy certain conditions, like one of them violating the KKT (Karush-Kuhn-Tucker) conditions. Equation 3 Compute the error for $a_{i}\;\mathrm{and}\;a_{j}\;$ and Calculate the error $E_{i}\;\mathrm{and}\;E_{j}$. for the selected Lagrange multipliers as shown in Equation 5:

$$E_{i}=f(x_{i})-y_{i}\;\mathrm{and}\;E_{j}=f(x_{j})-y_{j}\;$$ (5)

Where: $\left(x_{i}\right)$ is the prediction for sample $(x_{i}),y_{i}\;$ is the true label for $y_{j}$, $\left(x_{i}\right)$ is the prediction for sample $\left(x_{j}\right)$ and $y_{j}$ is the true label for $-y_{j}$ Select ***ai*** and ***aj*** to optimize: Choose ***ai*** and ***aj*** based on a heuristic or optimization criterion. Typically, these values are chosen to maximize the step size during optimization. Compute the kernel and update α values: Compute the kernel function $K(x_{i}),(x_{j})$, which measures the similarity between $\left(x_{i}\right)$ and $\left(x_{j}\right)$. Then, update ***ai*** and ***aj*** using the optimization formula as shown in Equations 6 and 7:

$$ai^{\mathrm{new}}=aj^{\mathrm{old}}+y_{i}(y_{i}-\;y_{j})ai^{\mathrm{old}}-ai^{\mathrm{new}}$$ (6)

$$ai^{\mathrm{new}}=aj^{\mathrm{old}}+\frac{y_{i}(y_{j}-\;y_{j})}{k(x_{i},x_{i})+k(x_{j},x_{j})-2k(x_{i},x_{j})}\;$$ (7)

Apply bounds: Ensure that the updated *α* values satisfy certain bounds: and **0** ≤ ***ai*** ≤ ***C*** and **0** ≤ ***aj*** ≤ ***C*** where *C* is the regularization parameter. Equation 7 Determine the threshold (*b*): After you've optimized the values, compute the threshold and Confirm convergence: Repeat steps 2–7 until convergence, which is commonly defined as when no more values can be optimized. The equations supplied here provide an overview of the essential steps of the SMO algorithm. Additional optimizations and details may be included in the real implementation to handle diverse cases effectively (51, 52).

### 3.4 Workflow of the proposed platform

**Figure 3:**
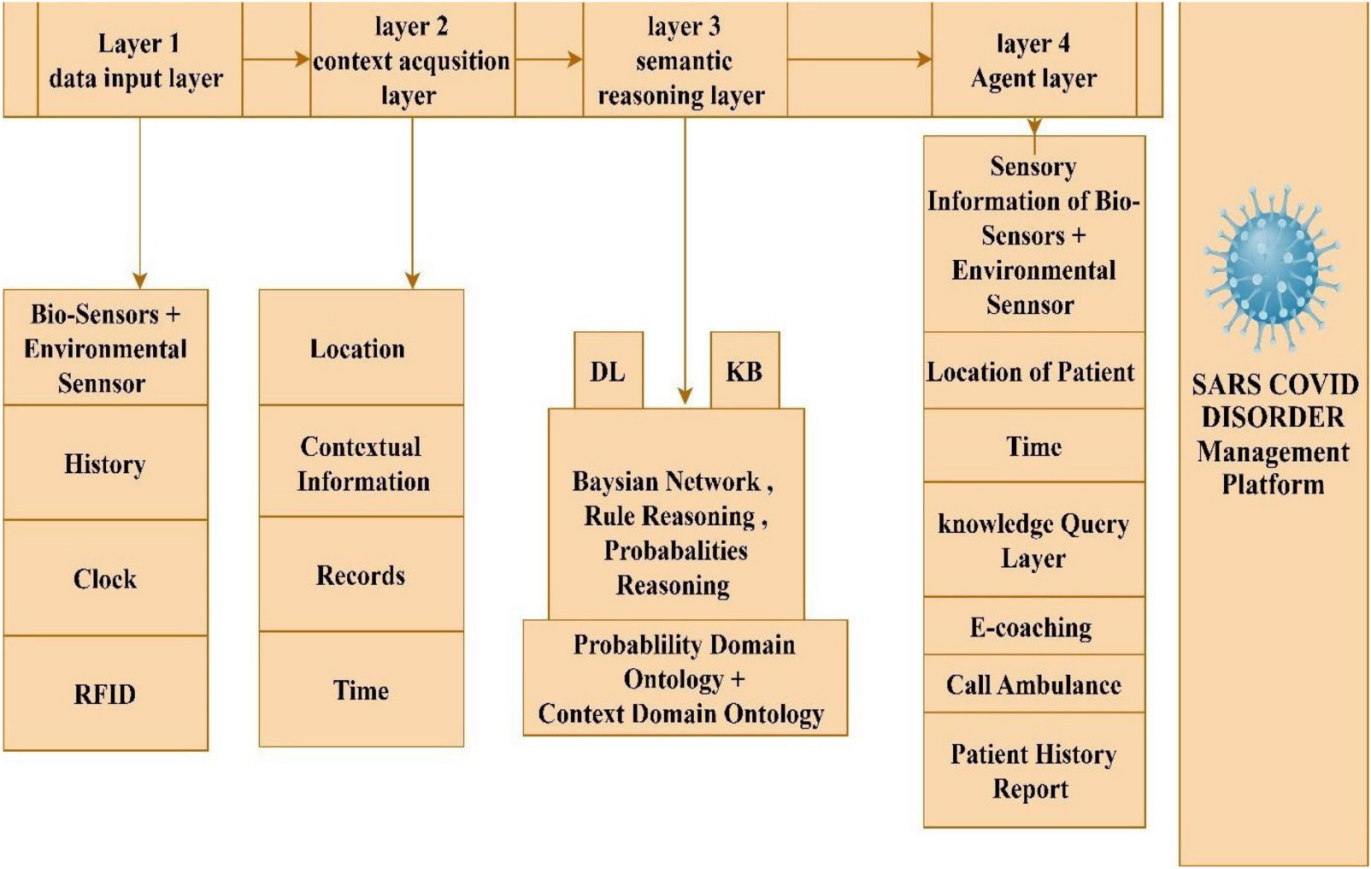
Proposed SARS COVID disorder management system.

The perception layer collects data, which is subsequently transformed and mapped to the Smart Rules by the platform. The rule engine communicates with the BBN, offers more evaluations, when necessary, determines appropriate reactions based on probability thresholds, and assures reaction efficacy through feedback governance. The agent works as ago-between for the system and the patient, enabling communication and carrying out activities (Figure 3). By using probabilistic reasoning and rule-based decision-making, the Platform intends to deliver intelligent, personalized monitoring and assistance for patients' health problems.

Naïve Bayes and Logistic Regression were intentionally selected due to their probabilistic interpretability, computational efficiency, and clinical traceability, which are critical requirements for real-time, context-aware medical monitoring systems. These lightweight models provide transparent decision boundaries and well-calibrated probability estimates, enabling clinicians and system designers to directly interpret risk scores and alert triggers. In contrast, more complex models such as deep neural networks or ensemble methods introduce higher computational overhead and reduced explainability, which can hinder trust, traceability, and safety in clinical decision-support settings.

To further support methodological rigor, a Random Forest classifier was employed as a baseline comparator. While Random Forests can capture non-linear relationships, their marginal performance gains did not outweigh the benefits of simpler probabilistic models in terms of interpretability, reproducibility, and seamless integration within the multi-agent simulation framework.

#### 3.4.1 Case study

**Figure 4:**
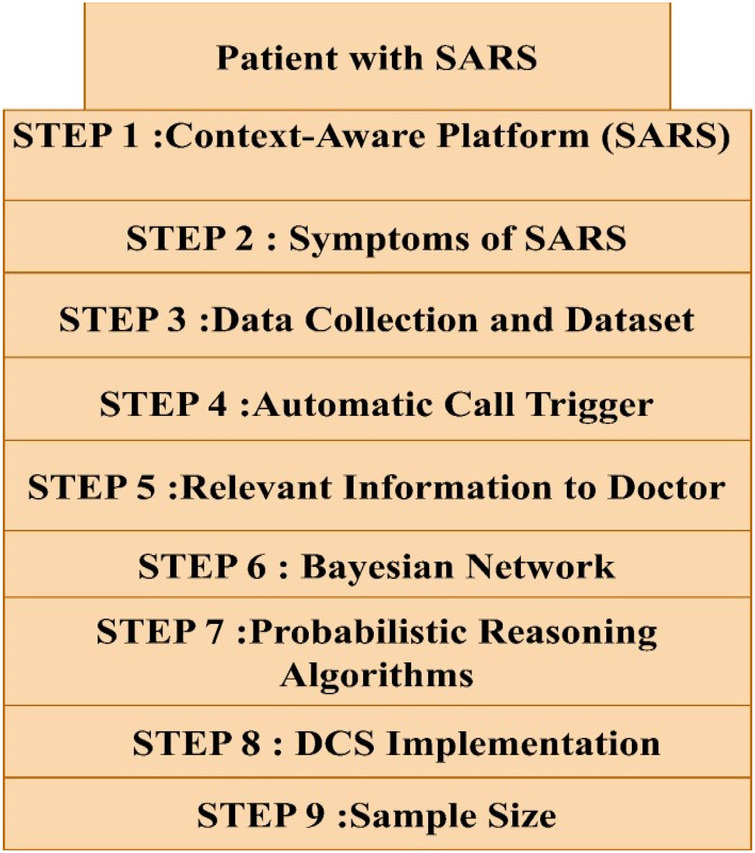
Patient treatment flow.

A SARS (severe acute respiratory syndrome) patient is admitted to the hospital in this case study, and a Context-Aware Platform (DCS) system is used to monitor the patient's status utilizing sensors, electronic equipment, and ambient elements (53–56) (Figure 4). SARS symptoms include shortness of breath and a drop in blood oxygen saturation (SPO_2_), which can lead to respiratory and cardiac failure. To diagnose a patient with SARS, the method uses particular parameters such as SPO_2_, body temperature, blood vessel pressure of carbon dioxide (PaCO_2_), and white blood cell count. A dataset is created based on these symptoms to automatically generate calls when the patient shows SARS symptoms.

The data collection contains cases classified as SARS and non-SARS situations. In the non-SARS situation, an increase in temperature does not automatically prompt a call to the doctor. In the SARS scenario, however, calls to the doctor are automatically initiated when the patient's blood oxygen saturation, tachypnea, and white blood cell count reach particular requirements. The system gives the clinician with pertinent information such as the bed number, ward number, location, and patient history. A Bayesian network is used to simulate the chance of certain situations or episodes happening, taking into account the influence of causes or risk factors. The Bayesian network, which represents both identified and undiscovered medical disorders, aids in probabilistic reasoning for screening and monitoring. To make educated judgements, the network analyses the impact of condition/episode symptoms and circumstances (3). Based on different parameters such as employee location and call importance, probabilistic reasoning algorithms prioritize judgements and pick relevant staff members to handle the call. The Context-Aware Platform architecture is used to create the DCS system, which allows it to automatically generate context calls depending on equipment and sensor data, such as temperature spikes or excessive light intensity in a patient's room. These thresholds and triggers are determined by the DCS's individual deployment environment. Furthermore, Description Logics (DL) models and DL Reasoners are used to formally validate and discover any flaws in the framework (57–59).

#### 3.4.2 Multi-agent reasoning

In Problog, we created rules that describe agents and their starting facts for monitoring a variety of health-related metrics (such as room temperature, blood oxygen saturation, body temperature, heart rate, blood sugar, blood pressure, and ECG) as well as a fire sensor (Table 2). Let's take a closer look at what this code is doing:

- Agents and First Facts: The code creates numerous agents (Agent 1, Agent 2, and so on), each with its own sort of sensor (e.g., room temperature sensor, blood oxygen saturation sensor). These agents are in charge of tracking and providing data on the health indicators of a patient called “Abdullah” with the ID “1”. They also keep track of the patient's whereabouts, which might be in the ICU, ward, CCU, or room.
- Rules (R1–R36): For each agent, the code comprises a set of rules (R1, R2, etc.). These rules govern how the agents gather and analyses data. R1 specifies, for example, that the presence of a patient with a given patient ID suggests that the thing is a patient. R4 also outlines the parameters for detecting high blood oxygen saturation.
- Conditions and criteria: Each agent monitors a certain parameter and compares it to predetermined criteria. Agent 1 (Room Temperature Sensor, for example) determines if the room temperature is high or low based on predefined criteria (“35” for high and “15” for low). A specified situation will be communicated to an external entity by the agent.
- Tell Statements: “Tell” statements are used in several rules, such as R8 and R26. These comments appear to infer that.
- Current Status: Based on the criteria and situations stated, the code seems to update the “current status” of several health measures such as blood pressure, heart rate, and ECG.
- Fire Sensor: A “Fire Sensor” is also referenced in the code. It keeps track of the “current fire level,” which might be “high,” “low,” or “no danger.”

In summary, our Problog simulates a system with many agents monitoring and reporting various health data and fire danger levels for a patient named “Abdullah” in various places. When particular situations are satisfied, the rules and conditions described in the code govern when they should be sent to external entities. For our complicated SARS medical status monitoring system, we represented a monitoring system with probabilistic and logical reasoning capabilities (25).

**Table 2:** Logical programming.

Agents	Initial facts & Rules
Agent 1: room temperature sensor	Initial Facts:
	Patient_Name(‘Abdullah’)
	Patient_ID(‘1’)
	Has_Patient_ID(‘Abdullah’,‘1’)
	Location(‘ICU’)
	Room_Temperature(‘10’)
	Rules:
	R1: Patient(P) ∧ Has_Patient_ID(P,PID) → Patient(P)
	R2: Location(L) ∧ Has_Room_Temperature(L,RT) ∧ RT ≥ 35 → Room_Temperature_Status(RT,High)
	R3: Location(L) ∧ Has_Room_Temperature(L,RT) ∧ RT ≤ 15 → Room_Temperature_Status(RT,Low)
Agent 2: blood oxygen saturation sensor	Initial Facts:
	Patient_Name(‘Abdullah’)
	Patient_ID(‘1’)
	Has_Patient_ID(‘Abdullah’,‘1’)
	Location(‘ICU’)
	BOS_Level(‘80’)
	Rules:
	R4: Location(L) ∧ Has_BOS_Level(L,BOS) ∧ BOS ≥ 90 → Current_BOS_Level(BOS,High)
	R5: Current_BOS_Level(BOS,High) → Tell[Current_BOS_Level(BOS,High)]
Agent 3: body temperature sensor	Initial Facts:
	Body_Temperature(‘38C’)
	Has_Body_Temperature(‘Abdullah’,‘38C’)
	Rules:
	R6: Patient(P) ∧ Has_Patient_ID(P,PID) → Patient(P)
	R7: Patient(P) ∧ Has_Body_Temperature(P,Temp) ∧ Temp ≥ 39 °C → Has_Fever(P,High)
	R8: Has_Fever(P,High) → Tell[Has_Fever(P,High)]
	R9: Patient(P) ∧ Has_Body_Temperature(P,Temp) ∧ Temp ≤ 37 °C → Has_Hypothermia(P,High)
	R10: Has_Hypothermia(P,High) → Tell[Has_Hypothermia(P,High)]
	R11: 37 °C < Temp < 39 °C → Current_Body_Temperature(P,Normal)
Agent 4: heart rate sensor	Initial Facts:
	Heart_Rate(‘90’)
	Has_Heart_Rate(‘Abdullah’,‘90’)
	Rules:
	R12: Patient(P) ∧ Has_Heart_Rate(P,HR) ∧ HR ≥ 100 → High_Heart_Rate(P)
	R13: High_Heart_Rate(P) → Tell[High_Heart_Rate(P)]
	R14: Patient(P) ∧ Has_Heart_Rate(P,HR) ∧ HR ≤ 60 → Low_Heart_Rate(P)
	R15: 60 < HR < 100 → Current_Heart_Rate(P,Normal)
Agent 5: blood sugar sensor	Initial Facts:
	Blood_Sugar(‘85’)
	Has_Blood_Sugar(‘Abdullah’,‘85’)
	Rules:
	R16: Patient(P) ∧ Has_Blood_Sugar(P,BS) ∧ BS ≥ 150 → High_Blood_Sugar(P)
	R17: High_Blood_Sugar(P) → Tell[High_Blood_Sugar(P)]
	R18: Patient(P) ∧ Has_Blood_Sugar(P,BS) ∧ BS ≤ 70 → Low_Blood_Sugar(P)
	R19: 70 < BS < 150 → Current_Blood_Sugar(P,Normal)
Agent 6: ECG sensor	Initial Facts:
	ECG(‘0.9’)
	Has_ECG(‘Abdullah’,‘0.9’)
	Rules:
	R20: ECG ≥ 1.2 → Abnormal_ECG(P)
	R21: Abnormal_ECG(P) → Tell[Abnormal_ECG(P)]
	R22: ECG ≤ 0.6 → Low_Abnormal_ECG(P)
	R23: 0.6 < ECG < 1.2 → Current_ECG(P,Normal)
Agent 7: blood pressure sensor	Initial Facts:
	Blood_Pressure(‘120/80’)
	Has_Blood_Pressure(‘Abdullah’,‘120/80’)
	Rules:
	R24: BP ≥ 145/100 → High_Blood_Pressure(P)
	R25: High_Blood_Pressure(P) → Tell[High_Blood_Pressure(P)]
	R26: BP ≤ 100/60 → Low_Blood_Pressure(P)
	R27: 100/60 < BP < 145/100 → Current_Blood_Pressure(P,Normal)
Agent 8: fire detection sensor	Initial Facts:
	Current_Fire_Level(High | Low | No_Risk)
	Rules:
	R28: Current_Fire_Level(Fire) → Tell[Current_Fire_Level(Fire)]

### 3.5 Results of multi-agent reasoning

**Figure 5:**
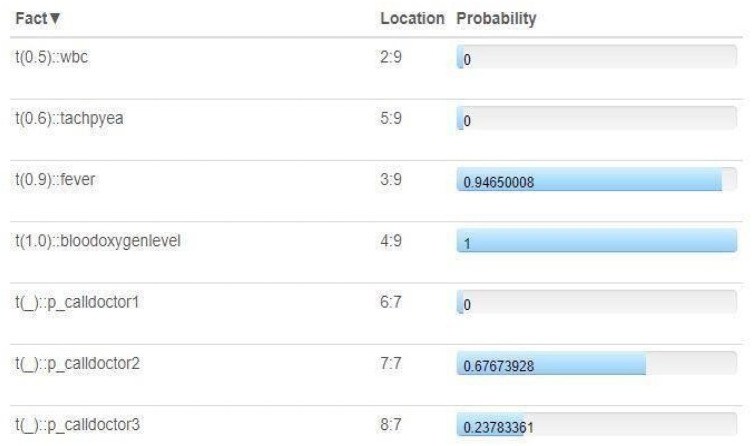
Multi agent reasoning.

The results showcased in Figure 5 show probabilistic assessments derived from sensor data and patient information, offering valuable insights into potential medical conditions and appropriate actions. Specifically:

- WBC (white blood cell count) and tachypnea (rapid breathing) exhibit varying degrees of uncertainty, indicated by probabilities of 0.5 and 0.6, respectively.
- The presence of fever is more certain, with a probability of 0.9.
- Blood oxygen levels are determined with high certainty, indicated by a probability of 1.0.
- Decision-making processes, such as the probability of calling the doctor, are influenced by multiple factors, resulting in probabilities ranging from 0.24 to 0.95.

These nuanced probabilities provide clinicians with valuable insights into the likelihood of specific medical conditions, aiding in informed decision-making and timely interventions. Such probabilistic assessments hold significant potential for enhancing patient care and optimizing healthcare delivery in real-time clinical settings.

#### 3.5.1 Multi-agent SARS management system simulation using NETLOGO

The structure and functions of a multi-agent healthcare system meant to monitor and manage patient health in a dynamic environment are simulated in this simulation. The system includes a variety of agents, including as patients, physicians, and sensors, each with its own set of tasks and duties. Patients are represented as turtle agents, who have properties including likelihood, patient profile, symptoms, communication, and medical treatments. Doctors: A new agent type specializing in medical skills. Blood oxygen, environmental, body temperature, heart rate, ECG, blood pressure, and room temperature sensors are examples of sensors. Probability Distribution Calculation: The simulation contains a “Calculate-Average-Probability” technique that calculates the average probability of all turtle agents. It loops through the agents, adds their probability, and computes the average. This simulation serves as the framework for a healthcare system in which agents work together to monitor patient health, execute medical operations, and respond to critical circumstances. The addition of sensor agents and the computation of average probability improve the system's decision-making skills, and the probability-based selection and instructions produced high probabilistic results by demonstrating system dependability and data consistency. Our suggested method simulation outperforms by assuring increased system performance, minimizing uncertainty and data inconsistency for multi-agent SARS management system.

This section may be divided by subheadings. It should provide a concise and precise description of the experimental results, their interpretation, as well as the experimental conclusions that can be drawn (8).

Pseudo/Algorithm Section 1: Agent Definitions and Setup {DEFINE Turtles-Own: Probability Patient-Profile Patient-Symptoms Patient-Communication Medical-Procedures is- Doctor}

Table 3 defines the core entities of the agent-based model, including patients, doctors, sensor agents, and environmental patches. Each agent is initialized with health-related attributes and behavioral flags to distinguish medical roles. Global variables are introduced to support probabilistic health assessment. The setup procedure initializes the simulation environment and resets all states for reproducible execution.

**Table 3:** Agent definitions and initialization (Section 1).

Component	Description
Agent type	Turtle agents (Patients, Doctors, Sensors) and Patch agents (Environment)
Patient attributes	Probability, Patient-Profile, Patient-Symptoms, Patient-Communication, Medical-Procedures
Doctor identifier	is-doctor (Boolean flag)
Patch attributes	Classification, Environmental-Factors, Medical-Equipment
Sensor agents	Blood-Oxygen Sensor, Environmental Sensor, Body-Temperature Sensor, Heart-Rate Sensor, ECG Sensor, Blood-Pressure Sensor, Room-Temperature Sensor
Global variables	total-probability, total-agents, average-probability
Initialization procedure	Clear simulation, create agents, initialize attributes, reset ticks
Movement procedure	Random agent movement (wander behavior)

##### 3.5.1.1 Pseudo/algorithm section 2: agent behaviors and critical conditions PROCEDURE GO

The main simulation loop governs agent interactions, movement, and health evolution over time. Patient probabilities are continuously updated while ensuring valid bounds as shown in Table 4. Critical health conditions are detected dynamically, triggering medical responses. The loop also updates visual indicators and advances simulation time through ticks.

**Table 4:** Core simulation loop and agent behaviors (Section 2).

Procedure	Functionality
Go	Executes the main simulation cycle
Behavior simulation	Updates agent behaviors and medical procedures
Probability management	Ensures probability values remain within defined bounds
Treatment handling	Manages ongoing and assigned medical treatments
Critical condition check	Evaluates patient health states for emergency response
Doctor notification	Calls nearest doctor when critical conditions are detected
Ambulance dispatch	Triggers emergency services for life-threatening cases
Visual update	Updates agent colors to reflect health states
Time advancement	Increments simulation ticks
Sensor invocation	Calls all sensor agent procedures sequentially
Statistical update	Computes average probability across all agents

Table 5 explains the emergency and alert handling mechanisms integrated into the model. It outlines how critical, extremely critical, and life-threatening conditions trigger different response levels. Doctor notification, alert generation, and ambulance dispatch are modeled to reflect real-world healthcare escalation. This ensures timely and prioritized medical intervention.

**Table 5:** Emergency and alert handling procedures.

Procedure name	Trigger condition	Action performed
Call-Doctor	Patient condition is critical and doctor agents are available	Notifies or dispatches the nearest doctor agent
Generate-Alerts	Patient condition is extremely critical	Generates alerts or system notifications
Call-Ambulance	Patient condition is life-threatening	Contacts ambulance or emergency medical services

##### 3.5.1.2 Pseudo/algorithm section 3: sensor agent procedures and probability calculation procedures for sensor agents

Sensor agents simulate continuous physiological and environmental monitoring of patients as shown in Table 6. Each sensor captures a specific health or contextual parameter and updates the patient's condition accordingly. This modular design allows independent sensor evaluation and easy extensibility. Sensor readings directly influence health probability calculations. Table 7 shows procedure computes a global health indicator by aggregating probabilities from all active agents. It iterates through the population, summing individual probabilities and counting agents. The final average probability provides a population-level health metric. This value supports real-time monitoring and decision-making within the simulation.

**Table 6:** Sensor agent procedures (Section 3).

Sensor agent	Purpose
Blood-oxygen sensor	Monitors blood oxygen saturation levels
Environmental sensor	Detects environmental conditions affecting patient health
Body-temperature sensor	Measures patient body temperature
Heart-rate sensor	Tracks heart rate variations
ECG sensor	Records electrical activity of the heart
Blood-pressure sensor	Monitors systolic and diastolic pressure
Room-temperature sensor	Measures ambient room temperature

**Table 7:** Average probability calculation algorithm.

Step	Description
1	Initialize total-probability to 0
2	Initialize total-agents to 0
3	Iterate over all turtle agents
4	Accumulate each agent's probability into total-probability
5	Increment agent count
6	Compute average-probability = total-probability/total-agents (if total-agents >0)

Although the proposed framework does not employ explicit longitudinal learning models such as recurrent or state-space architectures, temporal behavior is implicitly captured through repeated simulation ticks and continuous context updates. Each agent continuously integrates current physiological measurements with historical and contextual information, enabling the system to reflect evolving patient states over time. This design supports dynamic risk assessment and alert adaptation without requiring complex temporal model structures, aligning with the framework's emphasis on interpretability and real-time responsiveness.

### 3.6 System evaluation

The machine learning classifiers were trained and evaluated using a stratified 70/30 train–test split, preserving the Normal and SARS-risk class distribution to mitigate bias arising from moderate class imbalance. To enhance robustness, experiments were repeated across multiple randomized splits, and results are reported as mean ± standard deviation (SD).

All models were implemented in the WEKA machine learning environment. Logistic Regression employed L2 regularization using WEKA's default ridge parameter, with optimization constrained by a fixed maximum iteration limit to ensure convergence stability and prevent coefficient inflation. No feature weighting or polynomial expansion was applied, enabling direct interpretability of model coefficients. Naïve Bayes was implemented using probabilistic density estimation derived from observed feature distributions under the assumption of conditional independence; kernel density estimation was disabled to avoid overfitting and maintain calibration consistency across datasets.

Hyperparameter tuning was intentionally limited to preserve model transparency, reproducibility, and clinical traceability, which are essential for medical decision-support systems. Rather than exhaustive grid search, model stability was assessed through repeated stratified splits, ensuring that performance was not dependent on a single data partition.

Performance evaluation employed a comprehensive set of metrics, including accuracy, precision, recall, *F*1-score, Matthews Correlation Coefficient (MCC), ROC-AUC, and PR-AUC. Classification thresholds were selected using Youden's index to balance sensitivity and specificity in clinically relevant scenarios.

For statistical comparison, the best-performing model (Logistic Regression) was evaluated against Naïve Bayes using McNemar's paired test on matched test predictions, confirming that observed performance differences were statistically significant (*p* < 0.05). Metric variability across runs was quantified using standard deviation analysis, and the consistency of confidence intervals across simulation-based and external validation datasets was interpreted as evidence of generalization robustness.

Strict separation between training and testing data was enforced across all simulation runs and external validation experiments, ensuring the absence of information leakage. No samples were reused across folds or iterations, guaranteeing unbiased performance estimation.

The *F*1 score is calculated using the harmonic mean of accuracy and recall. It achieves an excellent balance of accuracy and recall; the results are as excellent as near most to 1 value on other hand as worst near to 0. It's calculated as follows as shown in Equation 8:

$$F1=\frac{2\times \mathrm{Precision}\;\times \mathrm{Recall}}{\mathrm{precision}+\mathrm{Recall}}$$ (8)

Precision measures the model's ability to make accurate positive predictions. It is calculated by dividing the total number of True Positives (TP) and False Positives (FP) by the number of True Positives (TP) the results are as excellent as near most to 1 value on other hand as worst near to 0 as shown in Equation 9:

$$\mathrm{Precision}=\frac{\mathrm{True}\;\;\mathrm{Positives}\;(\mathrm{TP})}{\mathrm{True}\;\;\mathrm{Positives}\;(\mathrm{TP})+\mathrm{False}\;\mathrm{Positives}\;(\mathrm{FP})}$$ (9)

It is a graphical representation of the performance of a binary classification model at various thresholds. It displays the True Positive Rate (sensitivity) vs. the False Positive Rate (1—specificity) at various categorization thresholds. The ROC curve's AUC (Area under the Curve) evaluates the model's overall performance. AUC of 1 indicates a perfect model, whereas AUC of 0.5 indicates a random classifier. A higher AUC value frequently indicates a more effective cancer detection algorithm. The Receiver Operating Characteristic (ROC) curve is a graphical representation of a binary classification model's performance, and the Area under the ROC Curve (AUC) is a single scalar value that represents the overall performance of the model. ROC Curve: Calculate TPR and FPR for various classification thresholds as shown in Equations 10 and 11:

$$F1=\frac{\mathrm{TP}}{\mathrm{TP}+\mathrm{FN}}$$ (10)

$$F1=\frac{\mathrm{FP}}{\mathrm{FP}+\mathrm{TN}}$$ (11)

TP: True Positives, FN: False Negatives, FP: False Positives, TN: True Negatives to build the ROC curve, plot TPR vs. FPR for each threshold.

The Matthews Correlation Coefficient (MCC) is a performance metric for binary classification models. True positives (TP), true negatives (TN), false positives (FP), and false negatives (FN) are all considered. The MCC formula is as follows as shown in Equation 12:

$$\mathrm{MCC}=\frac{\mathrm{TP}\;\times \;\mathrm{TN}\;-\;\mathrm{FP}\;\times \;\mathrm{FN}}{\sqrt{\left(\mathrm{TP}\;+\;\mathrm{FP})(\mathrm{TP}\;+\;\mathrm{FN})(\mathrm{TN}\;+\;\mathrm{FP})(\mathrm{TN}\;+\;\mathrm{FN}\right)}}$$ (12)

The number of correct positive forecasts is expressed as TP (True Positives). The number of correct negative predictions is expressed as TN (True Negatives). The number of false positives (FP) is the number of inaccurate positive predictions. The number of false negatives (FN) is the number of inaccurate negative predictions. The MCC scale is −1 to +1: A score of +1 denotes perfect classification, a score of 0 denotes no better than random classification, and a score of −1 denotes absolute disagreement between prediction and observation. MCC is especially beneficial when dealing with unbalanced datasets or when attempting to assess a model's performance in binary classification.

The Precision-Recall Curve (PRC) is a graphical assessment statistic that is used to evaluate the performance of a binary classification model, especially when dealing with unbalanced datasets. It illustrates the accuracy and recall levels at various probability thresholds. The precision equation mentioned above (Equation 12). Where TP (True Positives) is the number of positively predicted samples that were successfully predicted. The number of false positives (FP) is the number of positive samples that were mistakenly anticipated. The number of positive samples that were wrongly labelled as negative is denoted by FN (False Negatives). You compute accuracy and recall at different probability thresholds for categorizing positive examples to produce an accuracy-Recall Curve. Then you plot precision on the *y*-axis and recall on the *x*-axis to create a curve that demonstrates how the trade-off between accuracy and recall varies when the categorization threshold is changed.

External validation of the machine learning classifiers was conducted using the PhysioNet MIMIC-III Clinical Database (60), which provides multivariate physiological time-series data collected from intensive care unit patients. Relevant attributes including heart rate, ECG-derived features, body temperature, and blood pressure were extracted and preprocessed to align with the proposed system's input structure. Validation was performed in the WEKA environment to assess generalization beyond the simulation setting.

## 4 Results and discussion analysis

The experiment evaluated a number of machine learning algorithms, including Naive Bayes, Multinomial Naive Bayes, Decision Table, Logistic Regression, and SMO using physiological measurements of the EEG, heartbeat, blood pressure, body temperature, patient history, and system warnings. The measurement of performance was based on true positives (TP), false positives (FP), precision, recall, F-measure, Matthews correlation coefficient (MCC), receiver operating characteristic (ROC), and precision-recall curve (PRC). The performance of Naïve Bayes was satisfactory in terms of accuracy, recall, and F-measure, indicating that it is able to detect positive cases, however, the low value of MCC demonstrated that it is not suitable to handle imbalanced categories. Multinomial Naïve Bayes had a great TP and FP values and this demonstrated that it was proficient at identifying positive events but its ROC and PRC values showed that it did not possess much discriminative ability.

The performance of Decision Table was strong, with the matching high scores of precisions, recall, and F-measure, and average scores of MCC, ROC, and PRC, which indicated the good classification and discrimination. Strong accuracy, recall and *F*-measure were obtained by the Logistic Regression, supported by positive ROC and PRC values, which suggests high classification and an equal number of successes, which suggests an equilibrium in binary solutions. SMO also delivered quite stable results with relatively high precision, recall, and *F*-=, moderate MCC, and sufficient ROC and PRC values, thereby showing the effectiveness of the tool in the classification of instances and system stability. Together, these results support the importance of selective algorithm application in a non-monotonic system, based on context, to enhance the performance and reduce uncertainty and inconsistency of data.

Decision Table, Logistic Regression, and SMO algorithms provide strong classification and discriminatory features, which are essential to provide correct classification and make reasonable decisions. On the other hand, Naive Bayes and Multinomial Naive Bayes, though averagely good, have the limitation in dealing with imbalanced data. The present analysis shows that careful algorithm selection is the key to the creation of smart systems that can handle complex healthcare data and increase predictive accuracy and assist with the creation of reliable and data-driven clinical decisions to monitor and take care of patients.

Overall, the Decision Table and Logistic Regression algorithms stand out with reasonably good precision, recall, and *F*-measure scores, demonstrating their accuracy in identifying occurrences. These algorithms are also capable of discriminating, as evidenced by their ROC and PRC scores. However, additional criteria such as processing efficiency and interpretability may influence algorithm selection. It is critical to select appropriate algorithms to maintain system performance and minimize data uncertainty and inconsistency in the context-aware system. To modify and improve the system's performance, more study and comparisons with other algorithms or baseline models are recommended. The term SARS disorder is used in a generalized clinical sense to model severe respiratory distress patterns and should not be interpreted as a disease-specific or COVID-19 diagnostic framework.

Table 8 reports the averaged classification performance across repeated stratified train–test splits. Results are presented as mean ± standard deviation (SD) to reflect performance stability across randomized partitions. Logistic Regression achieved the highest overall performance, with an accuracy of 0.744 ± 0.018, precision of 0.748 ± 0.021, recall of 0.744 ± 0.019, *F*1-score of 0.743 ± 0.020, MCC of 0.530 ± 0.027, ROC-AUC of 0.820 ± 0.015, and PR-AUC of 0.827 ± 0.017.

**Table 8:** Evaluation results.

Algorithm	Attributers	TP	FP	Precision	Recall	*F*-measure	MCC	ROC	PRC
Naïve Bayes	ECG, heartbeat, class, historical-data, patient, room temperature, blood pressure, medical sensory, alert, blood oxygen saturation, body- temperature, System choice, probability.	0.654	0.393	0.653	0.654	0.641	0.238	0.780	0.800
Multinomial Naïve Bayes	ECG, heartbeat, class, historical-data, patient, room temperature, blood pressure, medical sensory, alert, blood oxygen saturation, body temperature, system choice, probability.	0.664	0.222	0.6999	0.564	0.6899	0.999	0.469	0.492
Decision table	ECG, heartbeat, class, historical-data, patient, room temperature, blood pressure, medical sensory, alert, blood oxygen saturation, body- temperature, system choice, probability.	0.729	0.221	0.799	0.729	0.723	0.533	0.732	0.721
Logistic regression	ECG, heartbeat, class, historical-data, patient, room temperature, blood pressure, medical sensory, alert, blood oxygen saturation, body- temperature, system choice, probability.	0.744	0.221	0.748	0.744	0.743	0.530	0.820	0.827
SMO	ECG, heartbeat, class, historical-data, patient, room temperature, blood pressure, medical, sensory, alert, blood oxygen saturation, body- temperature, System choice, probability.	0.722	0.231	0.786	0.722	0.716	0.512	0.746	0.693

To assess the statistical significance of performance differences, the best-performing model (Logistic Regression) was compared against Naïve Bayes using McNemar's paired test on matched test predictions. The analysis revealed a statistically significant difference in classification performance (*p* < 0.05), confirming that the observed improvements achieved by Logistic Regression are unlikely to be due to random variation.

**Figure 6:**
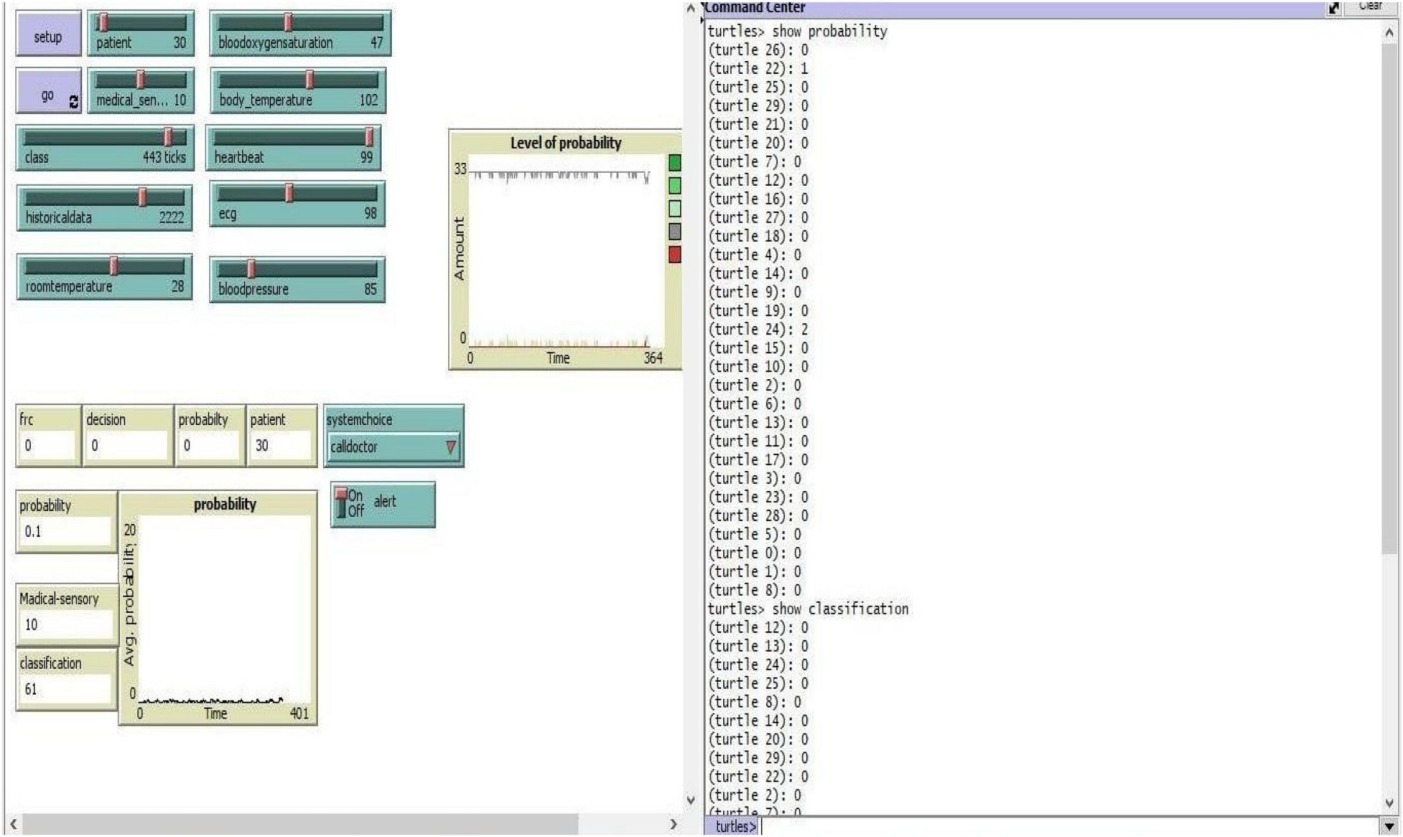
Behavior of multi agents in simulation.

The probabilistic outputs generated by the classifiers are transformed into clinically interpretable alert states through a structured thresholding mechanism (Figure 6). Predicted risk probabilities are mapped to three alert levels low risk (<0.3), moderate risk (0.3–0.6), and high risk (>0.6) based on empirical calibration and clinically motivated trade-offs between sensitivity and specificity. Threshold selection was guided by Youden's index to ensure balanced decision boundaries under class imbalance. These alert thresholds are configurable at the agent level, enabling adaptation to patient-specific risk profiles, monitoring intensity, and clinical context. Rather than acting as automated diagnoses, alert states are designed to support clinician-in-the-loop decision making, providing transparent probabilistic risk estimates alongside interpretable alert categories.

**Figure 7:**
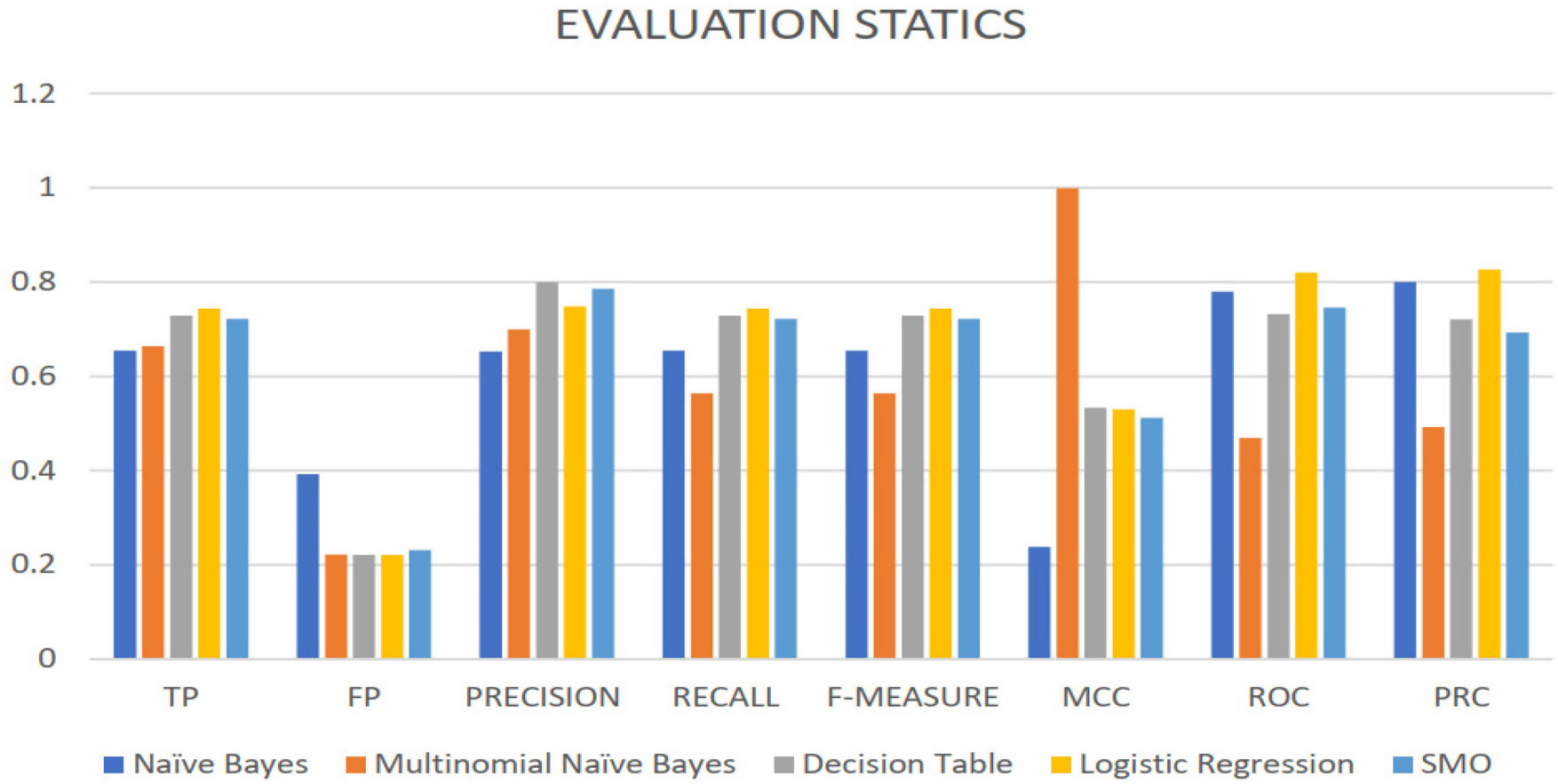
Evaluation statistics of ML models.

On the external PhysioNet MIMIC-III dataset, the proposed probabilistic classifiers demonstrated stable and consistent performance. Logistic Regression achieved an overall accuracy of 88.7%, with a precision of 0.87, recall of 0.89, *F*1-score of 0.88, and an ROC-AUC of 0.91. Naïve Bayes yielded a slightly lower but comparable accuracy of 86.2%, with balanced sensitivity and specificity across classes (Figure 7). These results closely align with the simulation-based outcomes, confirming the robustness and generalizability of the proposed classification framework.

The combined use of simulation-based evaluation and external dataset validation enables a dual-level assessment of the proposed framework. NETLOGO facilitates controlled experimentation of agent behavior, uncertainty propagation, and context-aware reasoning, while validation on the PhysioNet MIMIC-III clinical database confirms the robustness and generalizability of the underlying probabilistic classifiers. The close alignment of performance metrics observed across simulated and real-world physiological data supports the clinical plausibility of the proposed decision-support mechanism.

Although the machine learning classifiers were externally validated using the PhysioNet MIMIC-III clinical database with consistent and stable performance outcomes, the full end-to-end framework including multi-agent coordination, dynamic context updates, and alert dissemination was evaluated in a simulated environment. Consequently, real-world operational factors such as sensor noise, communication latency, heterogeneous sensor reliability, and clinical workflow integration remain outside the scope of the present validation.

Explicit longitudinal disease progression modeling was not implemented using recurrent or state-space learning architectures. Instead, temporal evolution is represented implicitly through sequential simulation ticks and context-aware state updates. While this approach enables continuous monitoring and adaptive reasoning, incorporating explicit temporal learning models to capture long-term disease trajectories remains an important direction for future work.

Future studies need to focus on optimizing these algorithms, hybrid models, and real-time context adaptation mechanisms to achieve the higher classification accuracy and system responsiveness especially in dynamic healthcare settings. Wearable devices, personalised treatment regimes, user-friendly applications and long-term follow-ups can enhance proactive care, and at the same time, guarantee ethical and legal standards. The interprofessional cooperation of artificial intelligence systems with the medical community and the strict clinical validation will additionally enhance the trust and help to become more widely accepted. However, the constraints remain, in particular, the challenge of dealing with highly unbalanced data and the complexity of scaling the system to a variety of real-life environments, which can affect the general consistency and performance.

## 5 Conclusion

Finally, the purpose of this study was to analyses the behavior of multi-agent in simulation as well as the performance of several machine-learning algorithms in a non-monotonic context-aware system with the goal of boosting system performance while minimizing data uncertainty and inconsistency. Nave Bayes, Multinomial Nave Bayes, Decision Table, Logistic Regression, and SMO were among the algorithms used. Performance indicators such as TP, FP, Precision, Re- call, F-Measure, MCC, ROC, and PRC were used in the evaluation. The Decision Table, Logistic Regression, and SMO algorithms got reasonably good precision, recall, and F-measure scores, showing their accuracy in identifying occurrences. The ROC and PRC scores revealed that these algorithms have strong classification capacity. Nave Bayes and Multinomial Nave Bayes, on the other hand, exhibited difficulty in dealing with unbalanced classes and had weaker discriminating skills. The study emphasized the need of selecting appropriate algorithms in a non- monotonic context-aware system to maintain system performance and minimize data uncertainty and inconsistency. The system can properly categorize cases and make optimum judgements based on the probability ratio by selecting relevant algorithms. The findings provide light on the performance of various algorithms in such a system and highlight the importance of careful attention when building and implementing context-aware systems. It is advised to investigate additional machine learning techniques or baseline models to improve the system's performance and address uncertainty. Furthermore, future research might concentrate on improving the system's design and introducing new features or data sources to improve decision-making. Our work advances the fields of non- monotonic reasoning, context-aware systems, and knowledge representation, notably in healthcare. The suggested architecture and assessment of various algorithms lay the groundwork for the development of intelligent monitoring and decision support systems that efficiently handle uncertainty and contribute to better patient care.

## Data Availability

The raw data supporting the conclusions of this article will be made available by the authors, without undue reservation.
